# Mediterranean Diet and Endothelial Function: A Review of its Effects at Different Vascular Bed Levels

**DOI:** 10.3390/nu12082212

**Published:** 2020-07-24

**Authors:** Jose D. Torres-Peña, Oriol A. Rangel-Zuñiga, Juan F. Alcala-Diaz, Jose Lopez-Miranda, Javier Delgado-Lista

**Affiliations:** 1 Lipids and Atherosclerosis Unit, Department of Internal Medicine, Maimonides Biomedical Research Institute of Cordoba (IMIBIC), Reina Sofia University Hospital, University of Cordoba, Av. Menéndez Pidal s/n, 14004 Cordoba, Spain; azarel_00@hotmail.com (J.D.T.-P.); oriol.rangel@imibic.org (O.A.R.-Z.); jlopezmir@uco.es (J.L.-M.); 2 CIBER Fisiopatología de la Obesidad y Nutrición (CIBEROBN), Instituto de Salud Carlos III, 28029 Madrid, Spain

**Keywords:** endothelial function, cardiovascular disease, Mediterranean diet

## Abstract

The Mediterranean diet has recently been the focus of considerable attention as a palatable model of a healthy diet. Its influence on many cardiovascular risk factors, combined with its proven effect in reducing the risk of cardiovascular events in primary prevention, has boosted scientific interest in this age-old nutritional model. Many of the underlying mechanisms behind its health-giving effects have been revealed, from the modulation of the microbiota to the function of high-density lipoproteins (HDL), and it seems to deliver its health benefits mainly by regulating several key mechanisms of atherosclerosis. In this review, we will review the evidence for its regulation of endothelial function, a key element in the early and late stages of atherosclerosis. In addition, we will assess studies which evaluate its effects on the functioning of different arterial territory vessels (mainly the microvascular, peripheral and central vascular beds), focusing mainly on the capillary, brachial and carotid arteries. Finally, we will evaluate the molecular mechanisms which may be involved.

## 1. Introduction

The Mediterranean diet has recently been the focus of considerable attention as a palatable model of a healthy diet. Its influence on many cardiovascular risk factors, as well as its proven effect on reducing the risk of cardiovascular events in primary prevention, with evidence from randomized controlled trials like the Prevención con Dieta Mediterránea (PREDIMED) study in Spain, has demonstrated that the Mediterranean diet is an effective means of preventing cardiovascular disease (CVD) [1] and has boosted scientific interest in this age-old nutritional model. Although individual components of the Mediterranean diet such as nuts, fish, olive oil and vegetables may be responsible for some of the effects of the diet, we believe that it is in the combination of the wide, complex variety of different nutrients where its main benefits lie. There is no single definition of the Mediterranean diet due to the variations in culinary traditions around the Mediterranean basin and their different adaptations around the world, and these variants may even modulate the influence of this diet on health [2]. In broad terms, however, the concept of the Mediterranean Diet is one of a diet relatively high in total fat, with at least 35% of calories from fat (mostly from olive oil), and low in saturated fats. The diet includes a wide variety of foods, and includes the use of olive oil for cooking and dressing; increased consumption of fruit, legumes and vegetables; low meat consumption, with white meat recommended over others (red or processed); an increased intake of fish; preparation of homemade sauces with tomato, garlic, onion with olive oil to dress rice, salads or other dishes; and a limited consumption of butter, fast food, sweets and sugar-sweetened beverages [3]. Many underlying mechanisms for its healthy effects have been shown, from the modulation of microbiota to HDL function, and it seems that the Mediterranean diet delivers its health benefits by regulating several key mechanisms of atherosclerosis, including endothelial dysfunction.

The endothelium is a complex, dynamic organ with multiple functions that maintains vascular homeostasis through various interactions between endothelial cells and the vascular lumen. Endothelial dysfunction is defined as an alteration in the physiology of the endothelium, characterized by an imbalance in the bioavailability of active substances of endothelial origin, which predisposes it to inflammation, vasoconstriction and increased vascular permeability, facilitating platelet aggregation, thrombosis and arteriosclerosis [4]. Endothelial dysfunction is a key element in the early and late stages of atherosclerosis, and it modulates the progression of plaque and the onset of atherosclerotic complications. In this review, we will cover the evidence of the effects of this diet on the regulation of endothelial function. This review draws from studies evaluating the function of different sized vessels from different vascular territories—in this case, the microvascular, peripheral and central vascular beds. To do this, we will mainly focus on the capillary, brachial, and carotid arteries, and end by evaluating the molecular mechanisms which may be involved.

## 2. Mediterranean Diet and Microvascular Endothelial Function

Microvascular endothelial function modulates the release of oxygen to cells. This mechanism is mainly controlled through humoral mediators, as the microvasculature lacks any anatomical structures which might allow nerve-mediated elasticity. Microvascular endothelial function is altered in several conditions, such as diabetes, hypercholesterolemia and hypertension [5,6], and evidence has shown how the treatment of cardiovascular risk factors improves microvascular endothelial function [7].

In this context, certain components of the Mediterranean diet, such as virgin olive oil, which is rich in monounsaturated fat and phenols, have been demonstrated to improve microvascular endothelial dysfunction. A study with hypercholesterolemic subjects found that the consumption of a meal containing virgin olive oil with a high phenolic compound content (400 ppm), compared to one with a reduced phenolic acid content (80 ppm), resulted in an improvement in microvascular endothelial function after ischemia in fasting and during the first 4 h of the postprandial period [8]. In another study, Valls et al. [9] showed that when virgin olive oil was replaced with a “basic” virgin olive oil enriched with phenols, this improved postprandial endothelial function—specifically ischemic reactive hyperemia—which increased after intake of this basic virgin olive oil from baseline to 5 h.

Some studies have combined the Mediterranean dietary pattern with other interventions. In a report from Fuentes-Jimenez et al. [10], 45 patients with metabolic syndrome followed a three-month intervention with a hypocaloric Mediterranean diet or a Mediterranean diet plus moderate-to-high-intensity training, to evaluate by laser-doppler how these two models modulate endothelial progenitor cell levels, cardiorespiratory fitness and microvascular endothelial function. The results showed that microvascular endothelial function improved in the Mediterranean diet plus moderate-to-high-intensity endurance training group compared to the Mediterranean diet alone group. Klonizakis et al. [11] investigated the effects of a combination of a Mediterranean diet and exercise on lower- and upper-limb microvascular functions in 22 older healthy subjects and observed that the Mediterranean diet group showed a greater improvement in endothelium-dependent vasodilation than the non-Mediterranean diet group. All of these reports support the idea that a Mediterranean diet may play a key role in the prevention of CVD through the modulation of microvascular endothelial function.

Moreover, a number of short-term interventions with a Mediterranean diet have also shown benefits for microvascular endothelial function. In a comparative pilot study with 24 young healthy participants who followed a one-month intervention, the effects of a Mediterranean diet and a vegan diet on microvascular function and plasma lipid levels in a healthy population were evaluated. In this study, the Mediterranean diet led to improvements in microvascular function [12]. All these findings have shed more light on the endothelial health-promoting benefits of the Mediterranean diet.

The elderly population may also gain benefit from a dietary intervention. In this context Marin et al. [13] evaluated in a crossover trial twenty elderly subjects who followed each of three different diets for one month: a saturated fatty acid diet; a Mediterranean diet enriched in monounsaturated fatty acids; and a low-fat diet. They found that ischemic reactive hyperemia was higher after consumption of the Mediterranean diet than with the other two diets.

In conclusion, microvascular endothelial function is impaired in populations with different cardiovascular risk factors. The evidence shows that the composition of the Mediterranean diet may modulate this impairment, improving the microvascular response (Table 1).

## 3. The Mediterranean Diet and Flow-Mediated Vasodilation of the Brachial Artery

Assessment of endothelial function using ultrasonography to evaluate the flow-mediated vasodilation (FMD) of the brachial artery is based on the fact that reduced blood flow in an artery induces a vasodilator response modulated by the endothelium [38]. Flow-mediated vasodilation is the gold standard technique to study endothelial function. It is especially interesting from a clinical point of view to evaluate the functionality of the vascular endothelium in the presence of certain clinical conditions such as diabetes, pre-diabetes or cardiovascular disease (carotid arteriosclerosis [39], peripheral vascular disease [40], coronary disease [41] and cerebral vascular disease [42]). It is important to note that this technique has a sensitivity of 71% and a specificity of 81% in predicting the existence of coronary heart disease [43]. Moreover, it is a non-invasive technique, in which each test costs little and allows researchers to evaluate the intervention easily, either with drugs, food, dietary models or physical activity.

One of the most relevant works to evaluate the influence of the Mediterranean diet over endothelial dysfunction was a sub-study of the CORDIOPREV trial (CORonary Diet Intervention With Olive Oil and Cardiovascular PREVention) conducted by our group [44]. In this work [14], endothelial function was studied in more than 800 participants by ultrasonography of the brachial artery and by calculating FMD at baseline and after 18 months of intervention with a Mediterranean Diet or a low-fat diet. The participants were categorized as patients with type 2 diabetes, pre-diabetes or without diabetes, according to the American Diabetes Association criteria. In this report, the Mediterranean diet increased FMD in patients with diabetes and pre-diabetes compared to baseline studies. Additionally, the Mediterranean diet improved FMD after 1.5 years compared to a low-fat diet in patients with diabetes. This study therefore supports the hypothesis that long-term consumption of a Mediterranean diet improves endothelial dysfunction, specifically in patients with pre-diabetes and diabetes with previously established atherosclerotic cardiovascular disease, and that it may modify the natural history of CVD in these subgroups of patients.

Schwingshackl and Hoffmann published a meta-analysis, including seventeen trials with 2300 participants, which showed that a Mediterranean diet improved inflammation markers (c-reactive protein (CRP) and IL-6) and FMD (weighted mean differences of 1.86%). These findings related to the changes in FMD are relevant because the meta-analysis suggested that for every one percent decrease in FMD, the future risk of a CVD-related event increased by 13% [45]. Specifically, the data provided by this meta-analysis indicated that the reduction of CVD risk in the Mediterranean diet groups is around 24% higher than in the control groups [46].

A recent meta-analysis [47] evaluated fourteen articles reporting data from 1930 participants, with study lengths ranging from four weeks to two years, in different target populations such as healthy participants [16,48], people with diabetes [17,18], overweight or obese participants [15,19,49], patients with metabolic syndrome [20], patients with acute coronary syndrome [50], and participants with previous cardiovascular disease and coexistence of type 2 diabetes and pre-diabetes [14]. The analysis reported that, overall, the Mediterranean diet improves the functionality of the endothelium in adults, with an average improvement of 1.66% in FMD (absolute change). It is important to highlight that this meta-analysis found a positive association between the study duration and the improvements in the functional indices of endothelial function, suggesting that longer periods of consumption of a Mediterranean diet may maximize the effects of this dietary pattern on endothelial function and theoretically lead to reductions in CVD risk.

To sum up, FMD is the main technique used for studying endothelial function in the brachial artery and it allows dietary interventions to be evaluated easily, such as those based on the Mediterranean diet. Different meta-analyses have proven the benefits of this dietary pattern in modulating endothelial function in different cardiovascular risk populations. Moreover, randomized control trials suggest that the improvement in endothelial dysfunction attributed to the Mediterranean diet could be potentially translated into a reduction in cardiovascular events, although these results need to be confirmed by further studies.

## 4. Mediterranean Diet, Intima-Media Thickness and Atherosclerotic Plaques

Carotid intima-media thickness is a marker of pre-clinical atherosclerosis and future cardiovascular events [51,52]. Observational reports have shown that carotid intima-media thickness is inversely related to adherence to dietary patterns with a high intake of vegetables and legumes and low in saturated fat-rich foods [53,54]. Over the last two decades, evidence from randomized trials has tested lifestyle interventions, such as diet, on carotid intima-media thickness progression rates with discordant results [21,22,23].

In the DIRECT-Carotid trial (Dietary intervention randomized controlled trial—carotid) study, 322 participants were assigned to three energy-restricted diets (Mediterranean, low-fat, or low-carbohydrate) and were followed for changes in the carotid wall. After two years of intervention, the researchers observed a 5% regression in mean carotid vessel wall thickness in all the intervention subgroups [21]. These results suggest that following a Mediterranean diet can cause a decrease in carotid atherosclerosis.

A report from the Northern Manhattan Study, which included 1374 participants, indicated that moderate and strict adherence to a Mediterranean diet may protect against a higher burden of carotid atherosclerotic plaques [22]. In this way, the reduction in intima-media thickness induced by the Mediterranean diet may mediate the different cardiovascular outcomes.

In a sub-study from the PREDIMED trial, researchers tested the effect of the Mediterranean diet on the progression of subclinical atherosclerosis in the carotid artery [23]. They evaluated 187 high-risk asymptomatic subjects assigned to three treatment arms: 66 participants to the Mediterranean diet with supplementary virgin olive oil; 59 participants to the Mediterranean diet with a supplement of nuts; and 62 to a control diet. Changes in mean intima-media thickness were measured after one year of intervention. Overall, no differences in intima-media thickness progression were observed after one year between the three groups. However, among participants with baseline intima-media thickness ≥0.9 mm, intima-media thickness after one year was reduced in the Mediterranean diet groups compared to the control group, which means that patients with a greater intima-media thickness benefit more from a dietary intervention than those with lower values for carotid thickness.

Later, another report from this group investigated the effects of both supplemented Mediterranean diets on intima-media thickness. They evaluated 164 subjects with completed data after an intervention of 2.4 years. Specifically, mean intima-media thickness progressed in the control group, but regressed in the Mediterranean plus nuts diet arm [24].

The benefits of the Mediterranean diet as regards carotid intima-media thickness have also been proved in certain subpopulations. Specifically, the study developed by Giannini et al. [25] evaluated the influence of a twelve-month Mediterranean diet on changes in lipid profile and carotid wall in hypercholesterolemic children. Interestingly, after the intervention period, a reduction in total cholesterol, LDL-cholesterol and intima-media thickness was achieved. This finding suggests that the Mediterranean diet is a valid way of treating hypercholesterolemia during childhood and improves long-term cardiovascular risk in this high-risk population. To evaluate the influence of adherence to the Mediterranean diet on carotid intima-media thickness and plaques in HIV-infected and non-HIV patients, Višković et al. [55] carried out a study in 110 HIV-infected and 131 non-HIV-infected participants. Their findings suggest that a lower adherence to the Mediterranean diet increased the risk of subclinical atherosclerosis and that HIV infection was a risk factor for subclinical atherosclerotic disease in older people.

Maiorino et al. [26] performed a parallel, two-arm trial in 215 participants with newly diagnosed diabetes to evaluate the long-term effects of a Mediterranean diet on carotid intima-media thickness and levels of endothelial progenitor cells, compared to a control low-fat diet. Compared to the control diet, the rate of regression in the carotid intima-media thickness was higher (51% vs. 26%) and the rate of progression lower (25% vs. 50%) in the Mediterranean diet arm. The consumption of a Mediterranean diet is also a valid approach for reducing carotid disease in patients with type 2 diabetes.

In conclusion, carotid intima-media thickness is a robust early marker of CVD progression. Both observational and randomized controlled trials suggest that the consumption of a Mediterranean diet can reduce the progression and induce regression of atherosclerotic plaques. It is important to highlight that a greater adherence to this pattern can modulate the effects on atherosclerotic plaques, supporting the mechanistic evidence for the reduction of cardiovascular events attributed to the Mediterranean diet.

## 5. Effect of Mediterranean Diet at Molecular and Cellular Levels

### 5.1. At the Molecular Level

#### 5.1.1. Mediterranean Diet and Genotype Relationship

Endothelial function is influenced by biological processes like glucose and lipid metabolism, and biochemical mechanisms such as inflammation, oxidative stress, cellular apoptosis or DNA damage, among others. The treatment and management of the disease focuses on controlling these processes and mechanisms. However, the effectiveness of the prevention tools and drugs used to treat the disease is conditioned by the genetic variability between individuals. Among the genetic elements that determine this response are single nucleotide polymorphisms (SNPs), which are a common genetic characteristic. SNPs are changes in the DNA sequence at the nucleotide level which can alter the sequence of genes that encode for specific proteins. These nucleotide changes in many genes may inhibit the synthesis of the protein for which the gene encodes, or may cause the synthesis of defective proteins which are involved in biological processes underlying the development of diseases. For this reason, these genetic characteristics of individuals have been proposed as biomarkers and have been studied to optimize drug treatments and lifestyle habits such as diet. Previous studies have shown an association between SNP and diet, as well as their relationship with biological markers of disease development [56].

One of the factors influencing endothelial function is the postprandial state [57]. Gómez-Delgado et al. studied the interaction between three genetic variants (*rs439401*, *rs440446* and *rs7412*) of apolipoprotein E (*APOE*), the long-term consumption of two healthy diets (a low-fat diet and a Mediterranean diet), and the effect of dietary intervention after three years on the postprandial metabolism of triglycerides in patients with CVD. The results showed that consumption of the Mediterranean diet in the long term induced, in the subjects carrying the T allele in the SNP *rs439401*, lower postprandial levels and smaller areas under the curve in triglycerides (TG), triglyceride rich lipoproteins (TRLs) and large TRLs than patients carrying the CC allele [27]. In this context, TG metabolism was also influenced by an interaction with the SNP *rs1800629*, located in the inflammation-related gene Tumor necrosis factor (TNF-α). Subjects with CVD carrying the G allele showed higher plasma fasting TG and C-reactive protein (hsCRP) levels in comparison with subjects carrying the A allele. The TG and hs-PCR plasma levels were lower in those who had a G variant after an intervention period of 12 months with a Mediterranean diet. These results suggest the Mediterranean diet could be used as a strategy to mute genetic characteristics associated with the risk of endothelial dysfunction [30].

In turn, one of the main characteristics of endothelial dysfunction is impaired nitric oxide (NO) bioavailability, as the result of low nitric oxide synthase (eNOS) activity, which is associated with the synthesis of pro-inflammatory molecules and reactive oxygen species (ROS). A previous study investigated the interaction between the presence of the NOS3 Glu298Asp polymorphism and the phenol content of virgin olive oil on postprandial endothelial dysfunction. The results showed that subjects with metabolic syndrome carrying the TT allele showed lower postprandial eNOS values, nitrate/nitrite ratio and maximum post-occlusive skin reactive hyperemia than subjects carrying the G allele. This effect was attenuated after the intake of an olive oil with a high content of polyphenols. Once again, according to this study, the intake of foods typical of the Mediterranean diet provides patients at risk of endothelial dysfunction with protection against disease-related genetic variants [31].

Another factor contributing to endothelial function is individual circadian rhythm [58]. A report by Corella et al. demonstrated the interaction of the Mediterranean diet and a SNP located in the *CLOCK* gene, a key component of circadian rhythms, on new cases of type 2 diabetes mellitus and CVD. The study showed that the Mediterranean diet increased the protective effects of the G allele against T2D and demonstrated the association between the G allele and protection against stroke in T2D subjects [28].

Finally, a growing body of evidence suggests that the Mediterranean diet may interact with genetic variants related to cellular aging. The aged cell, especially at the endothelial level, may also influence endothelial function. In this context, a study by Gomez-Delgado reported an interaction of the Mediterranean diet with the SNP *rs12696304,* located in the Telomerase RNA Component gene, which encodes the subunit of the telomerase enzyme, which is responsible for maintaining telomere stability (a biomarker of aging). The results demonstrated that the intake of a Mediterranean diet induced a protective capacity in the subjects carrying the CC allele, which was proven to slow down telomeric shortening and decrease hs-CRP levels in CVD patients [29].

#### 5.1.2. Effects of Mediterranean Diet on miRNAs

Epigenetic mechanisms regulate the expression and functions derived from the genetic information included in a person’s DNA. Processes like DNA methylation, methyltransferase activity and miRNAs control gene expression in reaction to environmental conditions, e.g., fatty acids, which may significantly influence the final genetic expression of a given product. In recent years, miRNAs have been considered powerful regulators of the genes involved in biological processes associated with the development of diseases such as nitric oxide homeostasis, inflammation or the cellular response to ischemia. These and other processes are related to endothelial function. The effect of diet and its components on miRNAs and their relationship to CVD have been investigated previously [59,60]. However, the study of miRNAs is relatively recent and their relationship with the Mediterranean diet is still poorly understood. In terms of the prevention of CVD, changes in miRNA expression in metabolic syndrome patients (*n* = 12) compared to people without the disease (*n* = 20) 4 h after the intake of 44 g of high polyphenol extra virgin olive oil (EVOO) were studied. The study showed that the intake of olive oil suppressed the expression of eight miRNAs (*miR-107, miR-769-5p, miR-192-5p, miR-15b-3p, miR19a-3p, miR-146b-5p, miR-548c-5p, miR-181b-5p*) and upregulated six miRNAs (*miR-1286, miR-619-3p, miR-302c-5p, miR-519b-3p, miR-614, miR-23b-3p*), expressed differentially between patients without disease and those with metabolic syndrome. The deregulation of these miRNAs by the acute intake of EVOO was associated with the beneficial effect of controlling disease-related biological processes and promoting an anti-inflammatory, anti-oxidant status, among other effects [32]. In another study, authors included 40 subjects with metabolic syndrome and analyzed changes in miRNAs and genes induced after an eight-week hypocaloric diet based on the Mediterranean dietary pattern. The results showed decreased expression of *miR-155-3p* and increased *Let-7b* in white blood cells as a consequence of the dietary treatment. The deregulation of these miRNAs has been previously associated with atherogenic mechanisms related to the development of diseases such as CVD [33]. Finally, in a more recent study including 12 healthy patients, the postprandial modulation of a group of CVD related-miRNAs after the intake of extra virgin olive oil with different polyphenol content (250, 500 and 700 mg total phenols/kg of oil) was investigated. The circulating levels of *let-7e-5p* decreased 6 h after the intake of the three oils, suggesting a polyphenol-independent effect. Also, higher postprandial levels of *miR-17-5p*, *miR-20a-5p* and *miR-192-5p* were observed after intake of the oils with low and medium polyphenol concentration. Deregulation of miRNAs in response to olive oil and polyphenols was associated with improved lipid metabolism and reduced oxidative stress. Thus, the regulation of miRNAs could constitute a strategy with benefits for endothelial function in response to food intake from the Mediterranean diet [34].

### 5.2. At the Cellular Level

#### 5.2.1. The Positive Impact of the Mediterranean Diet on Endothelial Progenitor Cells

Endothelial progenitor cells (EPC) play an important role, since the circulating cells are stem cells derived from bone marrow that can differentiate between the endothelial cells which cover and constitute the vascular endothelium [61]. Endothelial cell dysfunction leads to cellular apoptosis, a situation that requires the replacement of these cells. It is at this point where the circulating EPCs act as a defense mechanism by attaching themselves to the damaged vascular endothelium [62], thereby repairing its integrity and restoring its function [63]. The number of EPCs is reduced in some conditions like CVD [64], diabetes mellitus, and when there are multiple coronary risk factors. In fact, it has been proposed that atherosclerosis is caused by a consumptive loss of endothelial repairing capacity [65]. Previous studies have shown an increase in the number of circulating EPCs through statin drug therapy in patients with acute ischemic stroke [66]. Therefore, exogenous transplantation and strategies to increase the number of circulating EPCs have emerged as an alternative cell therapy for CVD. In the last 10 years, a number of studies have focused on the effect of the Mediterranean diet on circulating levels of EPCs in patients with CVD and those at risk of developing the disease. Fernandez et al., demonstrated in patients with metabolic syndrome that after 12 weeks of intervention, the number of EPCs (defined as CD34+KDR+) increased nearly two-fold with a Mediterranean diet and almost five-fold if the diet was coupled with physical activity. The study concluded that the Mediterranean diet improved the regenerative capacity of the endothelium and the cardiometabolic risk factors in MetS patients, mediated by an increase in EPC [10]. The beneficial effect on EPC was also observed at older ages. In line with the prevention of CVD, one study in a large cohort of nonagenarians (*n* = 421; mean age = 93.1 years) showed higher levels of EPC in subjects with high adherence to the Mediterranean diet, based on a Mediterranean diet score, than subjects with a lower adherence score. The study reported that the daily intake of Mediterranean foods like olive oil, fruit and vegetables was associated with higher levels of EPC than patients with low or no consumption of these foods [35]. Additionally, as previously mentioned in the section on microvascular endothelial function, one comparative study demonstrated that the consumption of a Mediterranean diet induced an increase in EPC in comparison with the intake of a saturated fatty acid or carbohydrate-rich diet in elderly subjects at risk of CVD. An increase in EPC levels was associated with an improvement in oxidative stress biomarkers and higher ischemic reactive hyperemia after the intake of the Mediterranean diet [13]. This improvement in circulating EPC levels was also shown in type 2 diabetic patients, after the Mediterranean diet, compared to the intake of a low-fat diet. This observation is directly related with intima-media thickness regression and differences in glycated haemoglobin, insulin resistance, total cholesterol, high-density lipoprotein cholesterol and systolic blood pressure levels, which were significantly better in the Mediterranean diet in comparison with a low-fat diet [26].

#### 5.2.2. Release of Microparticles Regulated by the Mediterranean Diet

A dysfunctional endothelium is characterized by an imbalance in nitric oxide production and consumption, in which there is high consumption and low production. This proinflammatory, pro-oxidant environment may cause cellular distress in the endothelium, affecting both blood circulating cells and endothelial cells. In this cellular pro-apoptotic stage, endothelial cells and platelets produce particles that are subsequently released into the plasma, as so-called microparticles. The microparticle membrane is made up of specific membrane proteins derived from its maternal cells and can be classified by flow cytometry into, for example, endothelial microparticles (EMPs), leukocyte microparticles (LMPs) and platelet microparticles (PMPs). The role of microparticles in the pathophysiology of CVD is still not fully understood, although it is evident that they form part of an intercellular communication system. Not only do the microparticles participate in the activation of inflammatory cells and the coagulation cascade, but they can also bind to target cells and activate diverse biological processes [67]. The most significant recent findings about microparticles are that their characterization and quantification makes them useful as biomarkers of the degree of endothelial dysfunction, and that they have been associated with a high risk of developing CVD. Changes in the release and number of microparticles can be modulated by aspects of lifestyle such as smoking or diet [68]. It is widely accepted that the first steps in managing and treating CVD risk involve changes in dietary and lifestyle habits; however, the effect of diet on the release of microparticles is only slight. In addition, previous studies within the framework of the PREDIMED study have demonstrated the regulatory effect of the Mediterranean diet on the release of microparticles. A case-control study including 25 subjects at risk of a cardiovascular event (non-CVE) and 25 who had suffered a CVE during follow-up showed that after one year of following a Mediterranean diet high in nuts, the levels of platelet-derived, endothelial-derived and activated cell-derived microparticles were lower in patients at risk of a cardiovascular event than patients who had suffered a CVE. In conclusion, the study showed that a Mediterranean diet rich in nuts may favorably modulate endothelial damage through the regulation of microparticle levels [36]. These results were in agreement with the findings of Marin et al., who reported that the concentration of total, endothelial and apoptotic microparticles decreased after four weeks of Mediterranean diet consumption in 20 elderly subjects at risk of CVD. The decrease in microparticle levels was associated with a reduced liberation of free radicals and less oxidative stress, due to the protective effect of the antioxidant components of the virgin olive oil in the Mediterranean diet [13]. Finally, a recent study including 155 subjects free of cardiovascular events but with a risk of disease showed, after an intervention with Mediterranean diets over one year, lower levels of procoagulant, platelet-derived and prothrombotic microparticles derived from activated cells compared to those on a low-fat diet. The study concluded that changes in the microparticles, which are biomarkers of cell activation and vascular damage, are modulated by the Mediterranean diet, inducing a less prothrombotic and procoagulant environment and delaying the development of CVD [37].

**Figure 1:**
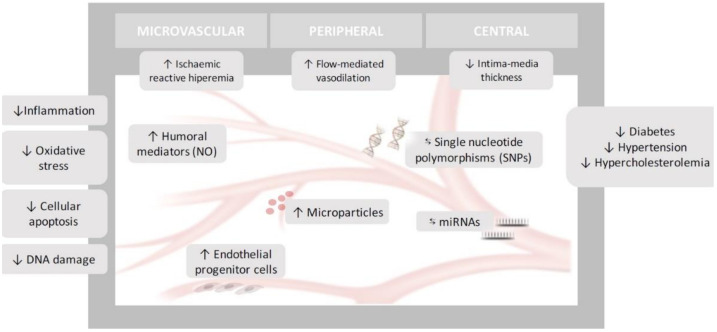
Effects of Mediterranean Diet on Endothelial Dysfunction. ↓: Decrease; ↑: Increase; ⇆: Interacts.

In summary, endothelial dysfunction encompasses a set of disorders that includes anatomical and functional disorders at different levels. In this work, we have explored how the Mediterranean diet has been linked to improvements in the endothelial function of different vessels of various sizes and from different vascular territories (microvascular, peripheral and central vascular beds) and how these effects are related to molecular changes and genetic regulation. Additional randomized clinical trials with larger sample sizes are needed to explore the long-term effects of the Mediterranean diet on endothelium functionality not only at the macro-microvascular level, but also at the molecular, genetic and epigenetic levels, thus allowing us to offer personalized dietary recommendations for the treatment and prevention of CVD (Figure 1).

## Figures and Tables

**Table 1:** Summary of the main characteristics of the trials investigating the effects of Mediterranean diet on endothelial function.

Authors (Ref)	Study Design	Population Health Status	Target	Sample Size	Duration	Type of Intervention	Type of Control	Main Findings
Fernandez et al. 2012 [10]	Parallel control trial	Metabolic syndrome patients	IRH by laser doppler	45	12 weeks	Mediterranean diet + moderate-to-high-intensity training	Hypocaloric Mediterranean diet	IRH was only improved after the Mediterranean diet plus training intervention.
Ruano et al. 2005 [8]	Crossover trial	Hypercholesterolemic	IRH by laser doppler	21	-	Olive oil (400 ppm of phenols)	Olive oil (80 ppm of phenols)	High-phenolic virgin olive oil improves IRH in the postprandium
Marin et al. 2011 [13]	Crossover trial	Healthy older subjects	IRH by laser doppler	20	4	Mediterranean diet	Saturated fat diet and a low-fat, high-carbohydrate diet.	IRH was higher after consumption of the Mediterranean diet compared to the two control diets (*p* < 0.05).
Klonizakis et al. 2013 [11]	Parallel	Healthy older subjects	CVC by laser doppler	22	8	Mediterranean diet + exercise	Non-Mediterranean diet + exercise	Mediterranean diet group showed greater improvement of endothelial function compared to non-Mediterranean diet group (*p* = 0.02)
Rogerson et al. 2018 [12]	Parallel	Healthy young people	CVC by laser doppler	24	4 weeks	Mediterranean diet	Vegan diet	Mediterranean diet led to improvements in microvascular function (*p* = 0.005)
Torres Peña et al. 2018 [14]	Parallel	Coronary heart disease with and without T2DM and pre-diabetes	Brachial artery FMD	805	1.5 years	Mediterranean diet	Low-fat diet	Mediterranean diet enhanced FMD in patients with diabetes (5.2 at 1.5 years vs. 3.8 at baseline; *p* = 0.04) and pre-diabetes (4.9 ± 0.4 vs. 3.8 ± 0.4; *p* = 0.04) Mediterranean diet induced an improvement in endothelial function after 1.5 years compared to low-fat diet in patients with diabetes (5.2 (Mediterranean diet) vs. 3.7 (Low-fat diet); *p* = 0.01)
Rallidis et al. 2009 [15]	Parallel	Healthy with abdominal obesity	Brachial artery FMD	90	2 months	Mediterranean diet plus close supervision	Mediterranean diet	The intervention increased FMD by 2.05%. Close adherence to a Mediterranean diet with dietitians’ advice improves endothelial function in obese individuals.
Davis et al. 2017 [16]	Parallel	Healthy older subjects	Brachial artery FMD	166	6 months	Mediterranean diet	Habitual diet	FMD was higher by 1.3% in the Mediterranean diet group.
Ceriello et al. 2014 [17]	Parallel	T2DM	Brachial artery FMD	24	3 months	Mediterranean diet	Low-fat diet	Mediterranean diet improved FMD compared to low-fat diet
Buscemi et al. 2009 [18]	Parallel	Overweight/obese	Brachial artery FMD	20	5 days and 60 days evaluation	Mediterranean diet	Very low-carbohydrate diet	Cardiovascular risk can be higher in the early days of a very low-carbohydrate diet compared to a Mediterranean diet. *p* = 0.007 for diet × time interaction
Jaacks et al. 2018 [19]	Parallel	Overweight/obese	Brachial artery FMD	30	8 weeks	Mediterranean diet. 3 arms: Mediterranean or habitual high-fat American-type diet + fish oil, walnuts and grape juice	High-fat American-type diet	No changes between groups were observed
Thomazella et al. 2011 [20]	Parallel	Stable coronary heart disease	Brachial artery FMD	40	3 months	Mediterranean diet	Low-fat Therapeutic Lifestyle Changes Diet	The 2 diets did not modify FMD in the brachial artery.
Shai et al. 2010 [21]	Parallel	Obese	IMT	140	2 years	Mediterranean diet; Low-carbohydrate diet	Low-fat diet	No differences in regression in intima-media thickness between groups.
Gardener et al. 2014 [22]	Observational	Primary and secondary prevention; multiethnic	IMT	1374	-	Mediterranean diet adherence score ranges.	-	Moderate and strict adherence to a Mediterranean diet may protect against a higher burden of carotid atherosclerotic plaque.
Murie-Fernández et al. 2011 [23]	Parallel	High-cardiovascular-risk asymptomatic subjects	IMT	187	1 year	Mediterranean diet + virgin olive oil and Mediterranean diet + nuts	Low-fat diet	Mediterranean diets + virgin olive oil or nuts were not effective in inducing regression IMT after one year. However, they were effective among subjects with high baseline IMT
Sala-Vila et al. 2014 [24]	Parallel	High-cardiovascular-risk asymptomatic subjects	IMT	175	2.4 years	Mediterranean diet + virgin olive oil and Mediterranean diet + nuts	Low-fat diet	Compared with a control diet
Giannini et al. 2013 [25]	Cross-sectional study	Hypercholesterolemic children	IMT	68	12 months	Mediterranean diet	-	A reduction in IMT was documented after intervention
Maiorino et al. 2016 [26]	Parallel	Newly diagnosed T2DM	IMT	215	8.1 years	Mediterranean diet	Low-fat diet	Mediterranean diet is associated with a reduction in the progression of subclinical atherosclerotic disease, compared to control diet
Gómez-Delgado et al. 2019 [27]	Intervention trial	Coronary heart disease patients	Genotyping of Apolipoprotein E genetic variants and the relationship with lipid metabolism	506	3 years; postprandial state after 4 h	Mediterranean diet	Low-fat diet	After long-term consumption of a Mediterranean diet, subjects carrying the T allele have lower postprandial levels and smaller AUC in triglycerides, TRLs and large TRLs than patients carrying the CC allele.
Corella et al. 2016. [28]	Longitudinal study	Free CVD patients (high-risk participants).	The study analyzed the relationship between CLOCK-rs4580704 gene variant and incidence of T2D and CVD.	7098	4.8 years	Mediterranean diet supplemented with extra virgin olive oil or Mediterranean diet supplemented with mixed nuts	Low-fat diet	The study showed that the Mediterranean diet increased the protective effects of the G allele against T2D and demonstrated the association between the G allele and protection against stroke in T2D subjects
Gómez-Delgado et al. 2018. [29]	Intervention trial	Coronary heart disease patients	Interaction between TERC gene variants with monounsaturated fatty acids and the effect on leukocyte telomere length, glucose metabolism and inflammation status	926	12 months	Mediterranean diet	Low-fat diet	After consumption of a Mediterranean diet, subjects carrying the CC allele have a protective capacity slowing down telomeric shortening and decreasing the hs-CRP levels in CVD patients
Gómez-Delgado et al. 2014 [30]	Intervention trial	Metabolic syndrome	Interaction between TNFα gene variation *rs1800629* and the Mediterranean diet. Effect on triglycerides and inflammatory markers	507	12 months	Mediterranean diet	Low-fat diet	The Mediterranean diet induced a silencing of a genetic variant of the TNF gene to improve the metabolism of triglycerides and the inflammatory markers associated with risk of endothelial dysfunction
Jiménez-Morales et al. 2011 [31]		Metabolic syndrome	Interaction between NOS3 Glu298Asp polymorphism and the phenol content of virgin olive oil. Effect on postprandial endothelial dysfunction	55	Postprandial state at 4 h	High, medium and low polyphenol extra virgin olive oil intake	---	Virgin olive oil with high content of polyphenols induces protection against the genetic variant NOS3 Glu298Asp, regulating NOS3 activity and decreasing the oxidative stress associated with endothelial damage.
D’Amore et al. 2016 [32]	Cross-sectional study	Metabolic syndrome	Deregulation of genes and microRNAs in response to extra virgin olive oil intake	12	Postprandial state at 4 h	High- and low-polyphenol extra virgin olive oil intake	----	The study showed the deregulation of miRNAs expressed differentially between healthy subjects and patients with metabolic syndrome. The deregulation of these miRNAs was associated with an anti-inflammatory, anti-oxidant status.
Marques-Rocha et al. 2016 [33]	Cross-sectional study	Metabolic syndrome	Effect of dietary strategy for weight loss on inflammation-related microRNAs	40	8 weeks	Hypocaloric diet based on the Mediterranean dietary pattern	----	Increased expression of *miR-155-3p* and decreased of *Let-7b* was observed in white blood cells. Changes in the expression profile were associated with atherogenic mechanisms related to disease development.
Daimiel et al. 2020 [34]	Crossover trial	Healthy subjects	Postprandial deregulation of miRNAs related to cardiovascular disease	12	Postprandial state at 6 h	Low, medium and high content of total phenols in extra virgin olive oil	----	Deregulation of miRNAs in response to olive oil polyphenols was associated with improved lipid metabolism and reduced oxidative stress
Fernandez et al. 2012 [10]	Parallel control trial	Metabolic syndrome patients	Effect of Mediterranean diet on endothelial progenitor cells (EPC) number.	45	12 weeks	Mediterranean diet plus moderate-to-high intensity endurance training	Hypocaloric Mediterranean diet	Mediterranean diet improved the regenerative capacity of the endothelium and the cardiometabolic risk factors in metabolic syndrome patients, mediated by an increase in EPC.
Cesari et al. 2017. [35]	Cross-sectional study	Elderly patients (nonagenarians)	Adherence of Mediterranean diet and endothelial progenitor cells (EPC) number.	421		Mediterranean diet	---	Strict adherence to the Mediterranean diet and a high consumption of its foods induces an increase in EPCs in elderly patients, suggesting a lower cardiovascular risk.
Marín et al. 2011 [13]	Crossover trial	Healthy older subjects	Effect of Mediterranean diet on biomarkers of regenerative capacity of endothelium	20	4 weeks	Mediterranean diet	Saturated fatty acid diet and low-fat, high-carbohydrate diet	The intake of the Mediterranean diet induced a decrease in microparticle levels and an increase in EPC, associated with an improvement in oxidative stress markers and high ischemic reactive hyperemia, suggesting an improvement in the regenerative capacity of the endothelium
Maiorino et al. 2016 [26]	Parallel	Newly-diagnosed type 2 diabetes patients	Effect of Mediterranean diet on EPC and IMT	215	4 years	Mediterranean diet	Low-fat diet	EPC levels increased with the Mediterranean diet and were associated with the regression of intima-media thickness and other markers of endothelial regeneration.
Chiva-Blanch et al. 2016 [36]	Case-control study	Patients with cardiovascular events and at high risk of a cardiovascular event	Microparticles released from different vascular cells as biomarkers of endothelial damage.	50	1 year	Mediterranean diet supplemented with virgin olive oil and Mediterranean diet supplemented with nuts	Low-fat diet	A Mediterranean diet rich in nuts promoted the reduction of endothelial damage markers, such as endothelial and platelet microparticles.
Chiva-Blanch et al. 2016 [37]	Prospective study	High cardiovascular risk participants free of cardiovascular events	Long-term effect of a Mediterranean diet on microparticles derived from blood cells	155	5 years	Mediterranean diet supplemented with virgin olive oil and Mediterranean diet supplemented with nuts	Low-fat diet	The Mediterranean diet induced changes in the microparticles, generating a lower prothrombotic and procoagulant endothelial environment and reducing the risk of cardiovascular events.

IRH: ischemic reactive hyperemia; CVC: cutaneous vascular conductance; FMD: flow-mediated vasodilation. IMT: intima-media thickness. EPC: endothelial progenitor cells; T2DM: type 2 diabetes mellitus; AUC: area under the curve; TRL: triglyceride rich lipoprotein; CLOCK: circadian locomotor output cycles protein kaput; TERC: Telomerase RNA component; hs-CRP: High sensitivity C-reactive protein; TNF: tumor necrosis factor; NOS3: Nitric Oxide synthase 3.
